# Frequent aberrant DNA methylation of *ABCB1, FOXC1, PPP2R2B *and *PTEN *in ductal carcinoma in situ and early invasive breast cancer

**DOI:** 10.1186/bcr2466

**Published:** 2010-01-07

**Authors:** Aslaug Aa Muggerud, Jo Anders Rønneberg, Fredrik Wärnberg, Johan Botling, Florence Busato, Jovana Jovanovic, Hiroko Solvang, Ida Bukholm, Anne-Lise Børresen-Dale, Vessela N Kristensen, Therese Sørlie, Jörg Tost

**Affiliations:** 1 Department of Genetics, Institute for Cancer Research, Oslo University Hospital Radiumhospitalet, Montebello, Oslo, N-0310, Norway; 2 Faculty of Medicine, Division The Norwegian Radium Hospital, University of Oslo, Montebello, Oslo, N-0310, Norway; 3 Department of Surgery, Uppsala University Hospital, Dag Hammarskjölds väg 20, Uppsala, SE-751 85, Sweden; 4 Department of Genetics and Pathology, Uppsala University Hospital, Dag Hammarskjölds väg 20, Uppsala, SE-751 85, Sweden; 5 Institute for Clinical Epidemiology and Molecular Biology (EpiGen), Faculty of Medicine, Division Akershus University Hospital, University of Oslo, Sykehusveien 27, Nordbyhagen, N-1474, Norway; 6 Department of Surgery, Akershus University Hospital, Sykehusveien 27, Nordbyhagen, N-1474, Norway; 7 Faculty of Medicine, Division Akershus University Hospital, Sykehusveien 27, Nordbyhagen, N-1474, Norway; 8 Biomedical Research Group, Department of Informatics, University of Oslo, P.O. Box 1080 Blindern, Oslo, N-0316, Norway; 9 Laboratory for Epigenetics, Centre National de Génotypage, CEA-Institut de Génomique, 2 rue Gaston Crèmieux, 91000 Evry, France; 10 Department of Biostatistics, Institute of Basic Medical Science, University of Oslo, P.O. Box 1122 Blindern, Oslo, N-0317, Norway

## Abstract

**Introduction:**

Ductal carcinoma *in situ *(DCIS) is a non-invasive lesion of the breast that is frequently detected by mammography and subsequently removed by surgery. However, it is estimated that about half of the detected lesions would never have progressed into invasive cancer. Identifying DCIS and invasive cancer specific epigenetic lesions and understanding how these epigenetic changes are involved in triggering tumour progression is important for a better understanding of which lesions are *at risk *of becoming invasive.

**Methods:**

Quantitative DNA methylation analysis of *ABCB1, CDKN2A/p16*^*INK4a*^, *ESR1, FOXC1, GSTP1, IGF2, MGMT, MLH1, PPP2R2B, PTEN *and *RASSF1A *was performed by pyrosequencing in a series of 27 pure DCIS, 28 small invasive ductal carcinomas (IDCs), 34 IDCs with a DCIS component and 5 normal breast tissue samples. *FOXC1, ABCB1, PPP2R2B *and *PTEN *were analyzed in 23 additional normal breast tissue samples. Real-Time PCR expression analysis was performed for *FOXC1*.

**Results:**

Aberrant DNA methylation was observed in all three diagnosis groups for the following genes: *ABCB1, FOXC1*, *GSTP1*, *MGMT, MLH1*, *PPP2R2B*, *PTEN *and *RASSF1A*. For most of these genes, methylation was already present at the DCIS level with the same frequency as within IDCs. For *FOXC1 *significant differences in methylation levels were observed between normal breast tissue and invasive tumours (*P *< 0.001). The average DNA methylation levels were significantly higher in the pure IDCs and IDCs with DCIS compared to pure DCIS (*P *= 0.007 and *P *= 0.001, respectively). Real-time PCR analysis of *FOXC1 *expression from 25 DCIS, 23 IDCs and 28 normal tissue samples showed lower gene expression levels of *FOXC1 *in both methylated and unmethylated tumours compared to normal tissue (*P *< 0.001). DNA methylation levels of *FOXC1, GSTP1, ABCB1 *and *RASSF1A *were higher in oestrogen receptor (ER) positive vs. ER negative tumours; whereas methylation levels of *FOXC1, ABCB1, PPP2R2B *and *PTEN *were lower in tumours with a *TP53 *mutation.

**Conclusions:**

Quantitative methylation analysis identified *ABCB1, FOXC1, PPP2R2B *and *PTEN *as novel genes to be methylated in DCIS. In particular, *FOXC1 *showed a significant increase in the methylation frequency in invasive tumours. Low *FOXC1 *gene expression in both methylated and unmethylated DCIS and IDCs indicates that the loss of its expression is an early event during breast cancer progression.

## Introduction

The multistep model of breast cancer progression suggests a transition from normal epithelium to invasive carcinoma via intraductal hyperplasia and *in situ *carcinoma [1]. These presumptive precursor lesions are currently defined by their histological features. Ductal carcinoma *in situ *(DCIS) is a pre-invasive lesion with diverse histological morphologies and molecular alterations [2]. The risk of DCIS progressing to invasive carcinoma is not well ascertained and robust biomarkers capable of stratifying the most aggressive from the more benign forms of the disease are currently lacking.

Cancer progression is due to the accumulation of genomic alterations leading to oncogene overexpression and tumour suppressor loss inducing growth advantage and clonal expansion. The transition of DCIS to invasive ductal cancer (IDC) is a poorly understood key event in breast tumour progression. Copy number alterations are already present in DCIS but their frequency tends to increase in IDCs [3]. Such genomic aberrations lead to altered gene expression, and comprehensive gene expression studies comparing DCIS and IDCs have identified stage-specific markers ([4-6] and Muggerud *et al*., submitted) along with a gene expression classifier which differed between DCIS and invasive breast cancer [7]. On the other hand, the frequency of *TP53 *mutations in DCIS is similar to what is observed in invasive tumours and *in situ *and invasive components from the same tumour exhibit the same mutations, indicating the same cellular origin of the two components [8-10].

Epigenetic changes are considered to be an early event during tumour development and one of the hallmarks of cancer [11]. Hypermethylation of CpG islands represents an alternative mechanism to inactivate tumour suppressor genes and is a prevalent early molecular marker for cancer. Specific patterns of CpG island methylation could result from clonal selection of cells having growth advantages due to silencing of associated tumour suppressor genes, DNA repair genes, cell-cycle regulators and transcription factors. Previous candidate gene studies investigated promoter hypermethylation of *in situ *lesions and identified aberrant methylation at the promoters of *GSTP1*, *CyclinD2*, *RARB2*, *Twist*, *RASSF1A, HIN-1, CDKN2A, 14-3-3σ *and *APC1 *[12-17]. However, only *GSTP1 *promoter hypermethylation was reported to progress in frequency during breast carcinogenesis [12].

Identification of early epigenetic changes in DCIS lesions might give valuable markers for early detection and may contribute to the understanding of how these changes affect the progression of the disease. The aim of this study was to identify informative progression markers by methylation analyses of eleven genes known to be methylated in breast tumours or breast cancer cell lines; *ABCB1 *[18], *CDKN2A/p16*^*INK4a *^[19], *ESR1 *[20], *GSTP1 *[21], *IGF2 *[22], *MGMT *[19], *MLH1 *[19], *PPP2R2B *[23], *PTEN *[24], *RASSF1A *[25] or displaying variation in breast cancer sub-type gene expression profiles; *FOXC1*.

In a series of 27 DCIS, 28 IDCs, 34 mixed cases (invasive tumours with *in situ *components) and 28 normal tissues we show that methylation of CpG islands is already detectable in DCIS with the same frequency as within IDCs.

## Materials and methods

### Patient material

Patients with fresh frozen tumour samples, collected at the Fresh Tissue Biobank at the Department of Pathology, Uppsala University Hospital, Sweden, were selected from a population-based cohort of 854 women diagnosed between 1986 and 2004, with either one of three types of primary breast cancer lesions; a) pure DCIS, b) pure invasive breast cancer, 15 mm or less, or c) mixed lesions (invasive carcinoma with an *in situ *component). All histopathological specimens, both paraffin embedded (used in IHC analyses) and frozen (used in methylation and quantitative real-time polymerase chain reaction (qRT PCR) analyses), were re-evaluated by a breast pathologist. Seventy-seven percent of the pure DCIS samples have a DCIS component of >70%. Seventy-six percent of the invasive samples have a tumour content of >70%, while 79% of the mixed samples have a tumour/DCIS component of >70%. DCIS lesions were classified according to the European Organisation for Research and Treatment of Cancer (EORTC) classification system [26]. We denoted the grades A to C (corresponding to grades I to III) to make clear that *in situ *and invasive lesions were classified based on different systems. Invasive breast cancers were classified based on the Elston-Ellis classification system, grades I to III [27]. Twenty-eight samples of normal breast epithelium were collected at the Akershus University Hospital from women undergoing a biopsy for the suspicion of malignant disease but without any histological findings. Five of the normal samples had enough DNA to be used in the methylation analyses of all genes in all patients. Twenty-three additional normal tissues were included in the methylation analysis of *FOXC1, ABCB1, PPP2R2B *and *PTEN *and qRT-PCR analyses of *FOXC1*. All patients signed a written consent to participate in the study, which has been approved by the regional ethical committee. Clinicopathological details of lesions are given in Table 1. This study was designed to investigate differences in CpG methylation events between different diagnostic groups with a particular emphasis on identifying specific markers related to tumour progression from *in situ *to invasive cancer. We would like to emphasise that this study was not designed to study prognosis. For example, none of the DCIS patients died from breast cancer and only three experienced a local recurrence. Also, the patients were treated differently according to tumour characteristics. Hence, no follow-up data can be presented. The study was approved by the Ethics Committee at Uppsala University Hospital (Dnr 2005:118).

**Table 1:** Clinicopathological factors

	No. DCIS	No. Invasive	No. Mixed
**Diagnosis**	27	28	34
			
**Oestrogen receptor status**			
Positive	19	23	24
Negative	8	4	8
			
**Progesterone receptor status**			
Positive	20	21	20
Negative	7	6	12
			
***TP53 *mutations**			
Wild type	22	23	24
Mutant	5	4	8
			
**Ki67**			
Positive	6	6	14
Negative	21	22	20
			
**Grade, I to III and/or A to C**			
Grade A/I (DCIS/invasive)	1	13	3/8
Grade B/II (DCIS/invasive)	13	10	8/17
Grade C/III (DCIS/invasive)	12	4	15/7

### Clinical endpoints

The entire coding sequence of the *TP53 *gene (exon 2-11) was analyzed for mutations by sequencing using the 3730 DNA Analyzer (Applied Biosystems, Foster City, California, USA) [10]. Immunohistochemical staining (IHC) of paraffin embedded material was performed for the oestrogen receptor (ER), progesterone receptor (PR) and a proliferation marker (Ki-67). A cut-off limit for positive staining was chosen for ER >10% (ER 6F11, Novocastra, Newcastle, UK), PR >10% (PR 1A6, Novocastra) and Ki-67 >10% (Ki-67 MIB-1, DAKO A/S, Glostrup, Denmark) stained tumour cells, irrespective of the intensity of the staining. Staining was performed in an automatic staining machine (Ventana Medical Systems, Tucson, AZ, USA).

### Pyrosequencing

A total of 1 μg of DNA was bisulphite converted using the MethylEasy™ HT Kit for Centrifuge (Human Genetic Signatures, North Ryde, New South Wales, Australia) according to the manufacturer's instructions. Quantitative DNA methylation analysis of the bisulphite treated DNA was performed by pyrosequencing or, in case of several sequencing primers, by serial pyrosequencing [28]. Oligonucleotides for PCR amplification and pyrosequencing were synthesized by Biotez (Buch, Germany) and sequences are given in Additional file 1. Quantitative DNA methylation analysis was carried out on a PSQ 96 MD system with the PyroGold SQA Reagent Kit (Pyrosequencing, Biotage, Uppsala, Sweden) and results were analyzed using the Q-CpG software (V.1.0.9, Pyrosequencing AB). Unmethylated commercial DNA (Qiagen, Valencia, CA, USA) and mixed human lymphocyte DNA (Promega, Madison, WI, USA) was analyzed in parallel to define the technical background. CpG-values for tumour and normal tissue samples are given in Additional file 2. Pyrograms for *FOXC1 *in six tumour samples and three normal tissue samples are given in Additional file 3.

### cDNA synthesis and real-time PCR analysis

qRT-PCR was performed on 25 DCIS, 23 pure invasive carcinomas from the same cohort and 28 normal tissues. cDNA was synthesized in a total volume of 20 μl with 100 ng total RNA using the High Capacity cDNA Reverse Transcription kit (Applied Biosystems,) and used as template for real-time PCR analysis with the TaqMan Gene Expression Assay for *FOXC1 *(Hs00559473_s1, Applied Biosystems,) on an ABI Prism 7900HT sequence detector system (Applied Biosystems). Universal human reference RNA (Stratagene, La Jolla, CA, USA) was used to generate standard curves. Each sample was run in triplicate. Relative gene expression levels were determined using the standard curve method and normalized to the reference gene *PGK1*. *PGK1 *was selected as a reference gene because the expression of this gene was not statistically significant different across the patient groups [29,30]

### Statistics

The average value of methylation for all CpGs in a target region was calculated for each sample and each gene. A sample was scored as aberrantly hypermethylated if the measured methylation values were two times above the standard deviation of the average of the normal controls, and conversely, as hypomethylated if methylation values were below two times the standard deviation of the average of the normal control tissues. Differences in the degree of methylation were determined by two-sided non-parametric Mann-Whitney test for two-categorical clinicopathological factors and Kruskal-Wallis test for three-categorical clinicopathological factors. The *P*-values were obtained by permutation procedure without any assumption on specific distribution. Correlations between concomitant methylated genes were calculated using Spearman's test. All tests were performed using the Statistical Package for Science version 15.0. For all obtained p-values, the false discovery rate (FDR) was assessed using Benjamini and Hochberg [31] in the Bioconductor multitest package [32].

## Results

### CpG promoter methylation patterns across three breast cancer diagnosis groups

**Figure 1:**
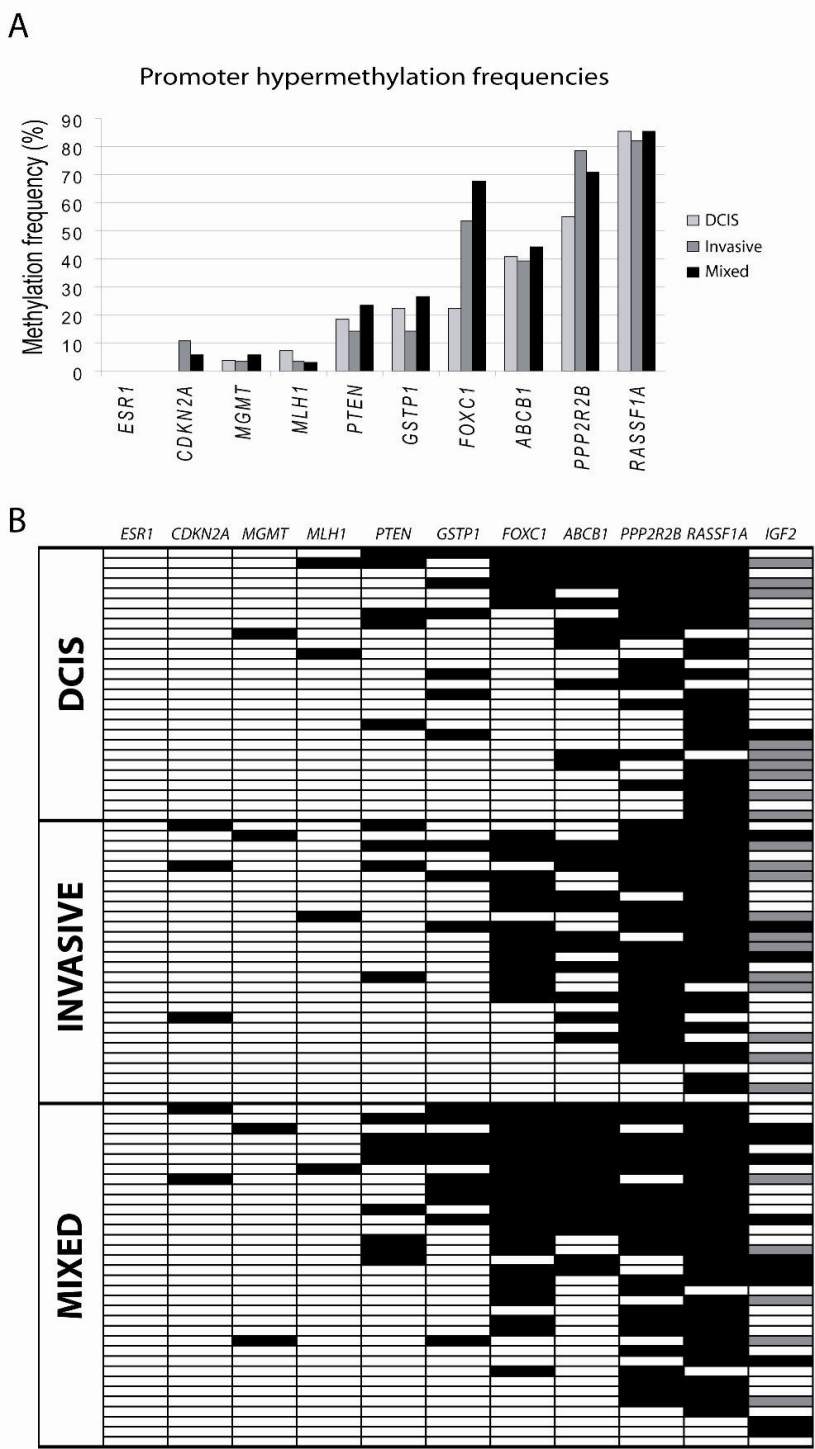
**Methylation overview**. **A: **Bar chart displaying promoter methylation frequencies across the three diagnosis groups. Methylation frequency is defined as the number of methylated samples within each category. The average values of methylation for all CpGs were calculated for each sample and each gene. A sample was scored as hypermethylated if the measured methylation values were two times above the standard deviation of the mean of the normal controls, and conversely, as hypomethylated if methylation values were below two times the standard deviation of the mean of the normal control tissues. DCIS = light grey, pure invasive = dark grey, and mixed = black. **B: **Methylation overview per gene across the three diagnosis groups. Black boxes indicate methylated and white boxes indicate unmethylated samples. For the imprinted gene *IGF2*; white boxes indicate the expected allele-specific methylation, black boxes indicate hypermethylation, and grey boxes indicate hypomethylation.

**Figure 2:**
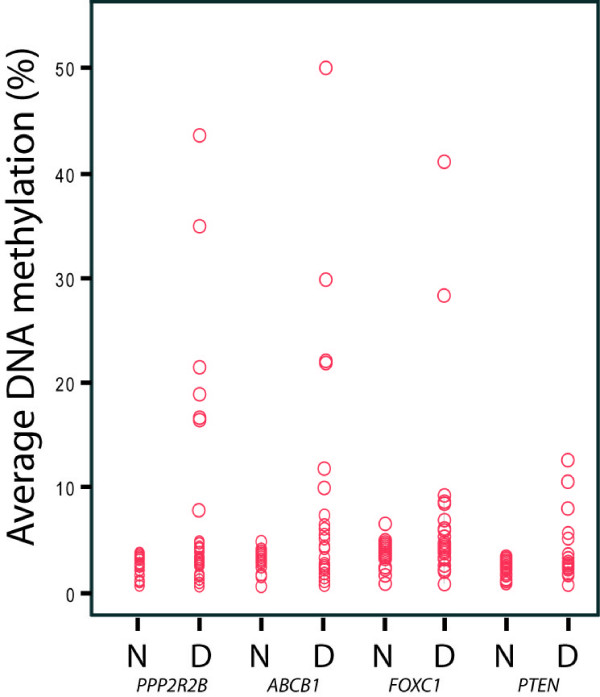
**Newly identified aberrantly methylated genes in DCIS**. Differences in the average DNA methylation (%) between normal and DCIS tissue for the newly identified methylated genes in DCIS; *PPP2R2B, ABCB1, FOXC1 *and *PTEN*. The average DNA methylation (%) value is the average value of methylation for all CpGs calculated for each sample. Abbreviations: N = normal tissue, D = DCIS.

**Figure 3:**
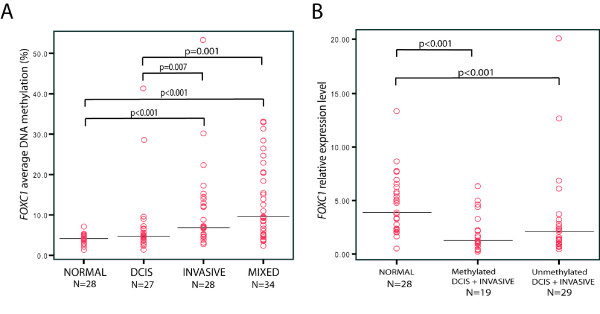
**Differential *FOXC1 *methylation across diagnosis groups and subsequent validation by qRT-PCR**. **A: **Differences in *FOXC1 *average DNA methylation (%) between normal breast tissue and the different diagnosis groups. The *FOXC1 *average DNA methylation (%) value is the average value of methylation for all CpGs calculated for each tumour sample. **B: **Differences in relative expression levels of *FOXC1 *as measured by qRT-PCR in normal breast tissue versus methylated and unmethylated tumours. Expression of *FOXC1 *was measured relative to the expression of the reference gene *PGK1*. Black horizontal bars represent median value for each diagnosis group.

The results of the pyrosequencing analysis of 27 DCIS, 28 invasive tumours and 34 mixed tumours, are illustrated in Figure 1A and 1B. All patient samples in all three groups displayed widespread aberrant CpG island methylation in the analyzed gene set. Aberrant DNA methylation (defined as an increase of average methylation levels beyond the average ± two times the standard deviation observed in the group of normal tissues) was observed in all three diagnosis groups for the following genes (frequency; % of DCIS vs. invasive vs. mixed): *ABCB1 *(40.7% vs. 39.3% vs. 44.1%), *FOXC1 *(22.2% vs. 53.6% vs. 67.6%), *GSTP1 *(22.2% vs. 14.3% vs. 26.5%), *MGMT *(3.7% vs. 3.6% vs. 5.9%), *MLH1 *(7.4% vs. 3.6% vs. 2.9%), *PPP2R2B *(55.0% vs. 78.6% vs. 70.6%), *CDKN2A/p16*^*INK4a *^(0% vs. 10.7% vs. 5.9%), *PTEN *(18.5% vs. 14.3% vs. 23.5%) and *RASSF1A *(85.2% vs. 82.1% vs. 85.3%). Methylation of *ABCB1*, *FOXC1, PTEN *and *PPP2R2B *in DCIS is reported here for the first time (Figure 2). To rule out any artefact due to inter-individual variation in *ABCB1*, *FOXC1, PTEN *and *PPP2R2B *methylation patterns in normal tissue and to confirm the presence of DNA methylation to be a tumour specific event, we analyzed the methylation patterns in 23 additional normal tissues without histopathological findings, confirming the initial findings of an absence of DNA methylation around the transcription start site of these genes in normal breast tissue (Figures 2 and 3A). Overall, methylation was observed already in DCIS and the frequency (that is, the number of methylated samples) was unchanged with the advancement of the disease for most of the genes except for *FOXC1*. The frequency of *FOXC1 *methylated samples increased from *in situ *to invasive cancer. In addition, differential levels of DNA methylation (average DNA methylation) of *FOXC1 *among the diagnosis groups were observed. For *FOXC1 *significant differences in methylation levels were observed between normal breast tissue and IDC (*P *< 0.001) and mixed tumours (*P *< 0.001) (Figure 3A). Significantly lower levels of *FOXC1 *methylation were observed in DCIS compared to invasive and mixed tumours (*P *= 0.007, and *P *= 0.001, respectively). No significant difference in DNA methylation levels between the diagnosis groups was observed for any of the other genes studied. The number of methylated genes was not different between DCIS, invasive and mixed patients. *ESR1 *did not show any difference in methylation levels between tumours and normal tissues. The imprinted gene *IGF2 *was hypomethylated in 10 DCIS, 11 invasive and 5 mixed tumours and hypermethylated in 1 DCIS, 3 invasive and 10 mixed tumours.

Concomitant DNA methylation was observed between genes at different chromosomes. Methylation at *FOXC1 *(chr 6p) showed significant correlations to *RASSF1A *(chr 3p), *PPP2R2B *(chr 5q), *ABCB1 *(chr 7q) and *GSTP1 *(chr 11q) (*P *= 7.6*10^-4 ^and R^2 ^= 0.34, *P *= 3.6*10^-5 ^and R^2 ^= 0.41, *P *= 4.5*10^-6 ^and R^2 ^= 0.46, *P *= 1.5*10^-4 ^and R^2 ^= 0.38, respectively). In addition methylation at *PTEN *(chr 10q) was significantly correlated to *ABCB1 *(chr 7q) and *PPP2R2B *(chr 5q) methylation (*P *= 1.4*10^-6^, R^2 ^= 0.48 and *P *= 1.0*10^-6^, R^2 ^= 0.60, respectively). All *P*-values were obtained with a false discovery rate <5%.

### qRT-PCR validation of the expression of *FOXC1*

The functional impact of *FOXC1 *methylation on its expression level was investigated by TaqMan qRT-PCR. Both methylated (n = 19) and unmethylated (n = 29) tumours showed significantly lower expression values compared to normal tissue (n = 28, both *P *< 0.001) (Figure 3B), indicating that the transcriptional inactivation of *FOXC1 *is an early event during tumour progression.

### Methylation profiles associated with clinicopathological features

**Figure 4:**
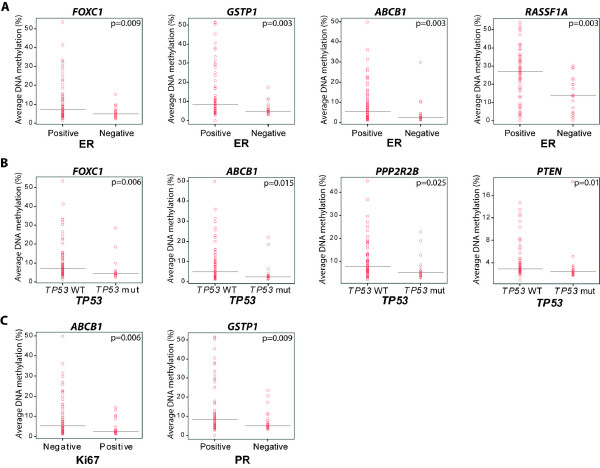
**Association between clinicopathological factors and DNA methylation**. **A: ***FOXC1, GSTP1, ABCB1 *and *RASSF1A *were significantly differentially methylated between ER-positive and ER-negative tumours. **B: ***FOXC1, ABCB1, PPP2R2B *and *PTEN *were significantly differentially methylated between *TP53 *wild type and mutated tumours. **C: ***ABCB1 *was significantly differentially methylated between Ki67-negative and Ki67-positive tumours and *GSTP1 *was significantly differentially methylated between PR-positive and PR-negative tumours. All types of lesions were combined for these analyses. All *P*-values were obtained by using a false discovery rate <5%.

Using the nonparametric Mann-Whitney test we found that *FOXC1*, *GSTP1*, *ABCB1 *and *RASSF1A *displayed a significantly different level of methylation between ER positive and ER negative samples (*P *= 0.009, *P *= 0.003, *P *= 0.003 and *P *= 0.003, respectively). Specifically samples with a negative ER status showed the lowest degree of methylation (Figure 4A). *FOXC1*, *ABCB1*, *PPP2R2B *and *PTEN *displayed significant differences in methylation levels in *TP53 *wild type and *TP53 *mutated samples (*P *= 0.006, *P *= 0.015, *P *= 0.025 and *P *= 0.01, respectively) with the *TP53 *mutated samples having the lower DNA methylation (Figure 4B). In addition *ABCB1 *methylation was lower in Ki67 positive tumours (*P *= 0.006) and *GSTP1 *methylation was lower in PR negative tumours (*P *= 0.009) (Figure 4C). No statistically significant difference could be attributed to histopathological grade.

## Discussion

In the present study we quantitatively determined the methylation levels in the promoter regions of 11 cancer-related genes in DCIS, small invasive breast cancers, mixed lesions and normal breast tissues. Aberrant DNA methylation was already present in DCIS for several of the genes studied. No DNA methylation changes specific for invasive breast cancer were identified.

Previous candidate gene studies have investigated promoter hypermethylation of *in situ *lesions and have shown methylation for *GSTP1*, *CyclinD2*, *RARB2*, *Twist*, *RASSF1A, HIN-1, CDKN2A, 14-3-3σ *[12-14,16]. We found frequencies of methylation for *RASSF1A *in both DCIS and invasive tumours similar to previously published reports ranging from 60 to 88% in different populations [14,15]. Results seem thus to be very consistent over different technology platforms identifying the epigenetic inactivation of *RASSF1A *as a very early step during breast carcinogenesis. *GSTP1 *has been found to be frequently methylated in different stages of breast carcinomas. Again, similar frequencies of 20 to 30% were found here for *GSTP1 *compared with recent reports [21,33-35]. In this study, methylation of *MGMT *was rare in both DCIS and invasive tumours, also in concordance with previous studies [36]. Infrequent methylation was also observed for the *MLH1 *gene, in line with the absence of changes in *MLH1 *expression during early breast carcinogenesis as assessed by IHC [37,38]. We also found minimal methylation of *CDKN2A *within the CpGs studied here, which is in concordance with a previous study on DCIS and other proliferative lesions of the breast [16]. No methylation of *ESR1 *was observed in this study, although we have used the commonly studied region 400 bp downstream of *ESR1 *transcription start site. A previous study observed methylation in DCIS samples [15], however they could not show significant differences between normal and diseased tissue in North-American and Korean populations. A study by Feng *et al*. (2007) shows the same result as we report with no increase in *ESR1 *methylation in malignant compared to normal breast tissues [39]. However, we can not exclude the possibility that the reason why we are not able to detect any *ESR1 *methylation is that pyrosequencing is less sensitive compared to the Q-MSP technology used in [15].

A novel finding of our study was the identification of aberrant DNA methylation of *ABCB1, FOXC1, PPP2R2B *and *PTEN *in DCIS. The methylation frequencies were similar in all diagnosis groups for *ABCB1, PPP2R2B *and *PTEN. PTEN *and *PPP2R2B *are both candidate tumour suppressor genes [23,40] and our results suggest that epigenetic silencing might be involved in dysregulation of these genes in DCIS. We have observed the methylation of *PPP2R2B *in locally advanced breast tumours (Dejeux *et al*, submitted for publication), and *PTEN *has been found to be frequently methylated in breast carcinomas [24,41]. *ABCB1 *is an ATP dependent p-glycoprotein involved in the efflux of various small molecules and xenobiotics in extra and intracellular membranes. Association of DNA methylation of *ABCB1 *and drug resistance in breast cancer cell-lines has been reported [42], and *ABCB1 *expression has been associated with poor outcome in breast cancer patients [43]. We have observed that *ABCB1 *methylation is important for treatment response and overall survival in patients with advanced breast cancer treated with doxorubicin (Dejeux E, *et al*. submitted). In this study, *ABCB1 *methylation was associated with non-proliferative, Ki67 negative tumours supporting a positive role for *ABCB1 *methylation in breast cancer progression and outcome. *FOXC1 *is a transcription factor with an important role in the regulation of ocular development [44]. *FOXC1 *is hypomethylated and highly expressed in CD44+ breast progenitor cells and might play an important role in the differentiation of mammary epithelial cell phenotypes [45]. In our data set, *FOXC1 *displayed a significantly increased methylation levels from normal breast tissue to invasive tumours with simultaneously lower *FOXC1 *gene expression as measured by qRT-PCR. The tumours with less methylation and somewhat higher expression of *FOXC1 *(Figure 3B) tended to be of the basal-like and normal-like breast cancer subtypes, as determined by gene expression profiling. Luminal B-like and ERBB2-like tumours had significantly lower *FOXC1 *expression (*P *= 0.026 and *P *= 0.018, respectively) compared to the basal-like tumours whereas no statistical significance was found for basal vs. luminal A-like tumours (*P *= 0.134) (Muggerud A *et al*., unpublished results). This supports the view of heterogeneous *FOXC1 *expression across molecular subtypes, which is in concordance with previously reported results [45]. Bloushtain-Qimron *et al *reported also increased methylation in matched distant metastases compared to the primary tumours, supporting a differential role for *FOXC1*-methylated cells in the progression of the disease. The 28 normal breast tissue samples analysed in this study displayed on average significantly higher levels of *FOXC1 *gene expression compared to the DCIS, small invasive and mixed lesions. Both the methylated and unmethylated DCISs and invasive tumours displayed significantly lower levels of *FOXC1 *gene expression. This might indicate that histone modifications or other mechanisms in addition to promoter hypermethylation silence *FOXC1 *in the unmethylated tumours. Further studies are needed to investigate the functional consequence of this increase of DNA methylation and the potential role of *FOXC1 *in the progression from DCIS to invasive carcinoma.

Concomitant DNA methylation was observed between some of the genes studied suggesting connected epigenetic programs within tumours. In line with our result, significant correlation between *GSTP1 *and *RASSF1A *hypermethylation has previously been reported [46]. The chromosomal region 6p25 harbouring the *FOXC1 *gene is frequently gained in ER negative tumours [47] in line with our observation of low DNA methylation and high expression in basal and normal-like tumours. The other chromosomal regions harbouring the genes with a high correlation to *FOXC1 *methylation have all been reported to either have gain or losses in breast cancer [48-50]. It is possible that concomitant DNA hypomethylation in these regions could induce chromosomal instability of the same regions as reported in colon cancer [51].

By unsupervised hierarchical clustering analysis of a number of methylation markers and tumours from 148 breast cancer patients, Widschwendter *et al*. (2004) [52], showed that the tumours segregated naturally into groups with distinct methylation profiles that differed significantly in their hormone receptor status. Further, other studies have focused on the epigenetic differences between ER positive and ER negative breast cancers and their results imply that methylation profiles of ER-positive tumours are different from those of ER-negative tumours [39,46]. Moreover they found that promoter hypermethylation of *RASSF1A *and *GSTP1 *was more frequent in ER-positive than in ER-negative tumours in both early and advanced breast tumours. Our data are consistent with these previous reports suggesting that ER (or hormone receptor) expression may influence epigenetic changes.

Lower levels of DNA methylation were observed in *TP53 *mutated tumours, especially at *FOXC1*, *ABCB1*, *PPP2R2B *and *PTEN *promoters. The association of *ABCB1 *and *PPP2R2B *with *TP53 *status is also observed in more advanced tumours (Dejeux *et al*. submitted). It has also been reported that breast tumours with *TP53 *mutations lacked methylation in a number of regulatory genes [39]. Further in concordance with our results, Toyota *et al*. [53] found a high number of *TP53 *mutations in unmethylated colorectal tumours suggesting that *TP53 *mutations and epigenetic alterations of other growth-suppressing genes can be two distinct mechanisms that inactivate tumour-suppressor genes in breast cancer. Similar to the results of [52] we could not find associations between histopathological grade and DNA methylation patterns among the samples and genes investigated. This might be due to independency of grade for this limited gene panel investigated or the relatively small cohort size.

Almost all genes methylated in DCIS and IDC have been identified using candidate gene approaches. Genome-wide methylation studies might provide useful in identifying new DNA methylation events occurring during early breast tumourigenesis. Since this study was designed to find differences related to tumour progression from *in situ *to invasive breast cancer, follow-up studies are needed to investigate these biological markers and their potential in predicting prognosis.

## Conclusions

This study has identified four novel genes as methylated in DCIS; *ABCB1, FOXC1, PPP2R2B *and *PTEN*. Their role in the progression from *in situ *to invasive carcinoma needs further investigation. Furthermore, these analyses demonstrate that promoter methylation is an early and frequent event in breast cancer and most of the genes that are found to be methylated in advanced breast tumours are already found methylated in DCIS.

## Abbreviations

DCIS: ductal carcinoma *in situ*; ER: estrogen receptor; FDR: false discovery rate; IDC: invasive ductal carcinoma; IHC: immunohistochemistry; PR: progesterone receptor; PCR: polymerase chain reaction; q-MSP: quantitative methyl-specific PCR; qRT-PCR: quantitative real-time PCR.

## Competing interests

The authors declare that they have no competing interests.

## Authors' contributions

AAM performed laboratory experiments, data analyses and wrote the manuscript. JAR performed laboratory experiments, data analyses and wrote the manuscript. FW was responsible for the patient cohorts, involved in the study design and wrote the manuscript. JB was responsible for the fresh frozen sample cohorts. FB and JJ were involved in laboratory experiments. HS was involved in the statistical analyses. IB provided normal breast tissue samples. ALBD, VNK, TS and JT initiated and designed the study and were involved in writing the manuscript. All authors have read and approved the final manuscript.

## Supplementary Material

Additional file 1 Additional file 1 is a table listing PCR and pyrosequencing primers. Click here for file

Additional file 2 Additional file 2 is a table listing CpG average methylation values. Click here for file

Additional file 3 Additional file 3 is an overview of pyrograms for *FOXC1 *in six tumour and three normal samples. Click here for file
