# Supplementary material for: Frequent aberrant DNA methylation of ABCB1, FOXC1, PPP2R2B and PTEN in ductal carcinoma in situ and early invasive breast cancer
Source: Breast Cancer Res. 2010 Jan 7;12(1):R3. doi: 10.1186/bcr2466 (PMC2880421; doi:10.1186/bcr2466)
Supplement: Additional file 3 — is an overview of pyrograms for FOXC1 in six tumour and three normal samples. [file bcr2466-S3.doc]

FW06-123

FW06-29

FW06-49

FW06-256

FW06-36

FW06-102

24

25

76
